# Screen-based sedentary behavior during adolescence and pulmonary function in a birth cohort

**DOI:** 10.1186/s12966-017-0536-5

**Published:** 2017-06-23

**Authors:** Bruna Gonçalves C. da Silva, Ana M. B. Menezes, Fernando C. Wehrmeister, Fernando C. Barros, Michael Pratt

**Affiliations:** 1 Postgraduate Program in Epidemiology, Federal University of Pelotas, Pelotas, RS Brazil; 2 Postgraduate Program in Health and Behavior, Catholic University of Pelotas, Pelotas, RS Brazil; 3 Institute of Public Health, Department of Family Medicine and Public Health, School of Medicine, University of California, San Diego, CA USA

**Keywords:** Adolescents, Epidemiologic studies, Lifestyle, Lung, Respiratory function

## Abstract

**Background:**

Adolescents spend many hours in sitting activities as television viewing, video game playing and computer use. The relationship between sedentary behavior and respiratory health remains poorly elucidated. To date there have been no studies evaluating the relationship between sedentary behavior and pulmonary function in young populations. The purpose of this study is to examine the association between the trajectory of screen-based sedentary behavior from 11 to 18 years and pulmonary function at 18 years in a Brazilian birth cohort.

**Methods:**

Data from a longitudinal prospective study conducted among the participants of the 1993 Pelotas (Brazil) Birth Cohort. Time spent on television, video games, and computers during a weekday was self-reported at ages 11, 15 and 18 years. For each age, sedentary behavior was defined as the sum of time spent on these screen-based activities. To evaluate the sedentary behavior trajectory during adolescence group-based trajectory modeling was used. Outcome variables were three pulmonary function parameters: forced expiratory volume in one second (FEV_1_), forced vital capacity (FVC), and peak expiratory flow (PEF), evaluated by spirometry, at 18 years expressed as z-scores. Crude and adjusted linear regressions, stratified by sex, were performed.

**Results:**

The three-group trajectory of sedentary behavior was the best fitting model. The trajectory groups were: always high (representing 38.8% of the individuals), always moderate (54.1%), and always low (7.1%). In the adjusted analyses, boys in the always-low group for sedentary behavior had higher FVC at 18 years (β = 0.177; 95% CI:0.027;0.327; *p* = 0.021) than boys in the always-high group. There were no differences for other pulmonary function parameters in boys. No significant association was found for girls.

**Conclusion:**

The trajectory of screen-based sedentary behavior throughout adolescence was not consistent associated with pulmonary function at 18 years.

**Electronic supplementary material:**

The online version of this article (doi:10.1186/s12966-017-0536-5) contains supplementary material, which is available to authorized users.

## Background

Sedentary behavior is commonly defined as time spent engaged in sitting or lying down that requires energy expenditure from 1.0 to 1.5 basal metabolic rates (METs) [1]. It includes activities such as television viewing and use of video games/computers [2]. Adults and adolescents spend many hours in sitting activities worldwide [3–7]. In a study of 66 countries with individuals aged 15 years it was observed that the overall proportion of individuals spending four or more hours per day sitting was 41.5% [4]. A population based study in the United States showed that 56% of adolescents between 12 and 15 years of age watched two or more hours of television per day [3]. A Brazilian population based study carried out in a representative sample of adolescents observed that 73.5% of the population from 12 to 17 years spent two hours or more per day on television viewing, video game playing, and computer use [5].

While physical activity is established as a protective factor for non-communicable diseases and mortality [8], several studies have shown a positive association between excessive time in sedentary behaviors and many negative health outcomes [9], including all-cause mortality [10–12], obesity [13], and development of non-communicable diseases [11, 14]. The negative impact of excessive sedentary behavior may be independent of the protective effect of meeting physical activity recommendations for some health outcomes [15].

Despite the rapid expansion of research on sedentary behavior and its health consequences, the relationship between sedentary behavior and respiratory health remains poorly elucidated. Most research on this subject has investigated asthma or wheezing as the primary outcome instead of measuring pulmonary function [16–20]. Previous studies have shown inconsistent results for the association between time spent on screen-based activities and asthma or asthma symptoms. While some cross-sectional studies with children and adolescents showed positive associations between time spent on sedentary behavior and asthma symptoms [16–23] or asthma [18, 24–26], other studies did not find significant cross-sectional associations [20, 27]. Vogelberg et al. [17] analyzed the longitudinal association between television viewing and computer use with development of wheezing in adolescents and did not find a significant association after stratifying the analysis for smoking status. On the other hand, Sherriff et al. [19] found an association between duration of television viewing in early childhood and developing asthma by age 11. Individuals with asthma do not necessarily have reduced pulmonary function in the absence of an asthma crisis [28].

To date there have been no studies evaluating the relationship between sedentary behavior and pulmonary function in young populations. However, pulmonary function is an important health outcome; a reduced pulmonary function is associated with increased risk of respiratory disorders, all-cause, cancer, respiratory, and cardiovascular mortality [29–31]. Also, individuals with reduced pulmonary function during childhood may have increased mortality risk in adulthood [32].

Therefore, the aim of this study was to examine the association between the trajectory of screen-based sedentary behavior from ages 11 to 18 years and pulmonary function at 18 years in a birth cohort from Brazil.

## Methods

The current study was carried out with participants from the 1993 Pelotas Birth Cohort. All hospital-born children in the calendar year of 1993 whose families were living in the urban area of Pelotas, a city located in the Southern of Brazil, were eligible to participate in the 1993 Pelotas Birth Cohort Study. Of the 5265 live births occurring in Pelotas in 1993, 5249 mothers (99.7%) agreed to participate and were interviewed in the hospital soon after delivery. Trained interviewers conducted the interviews on sociodemographic, behavioral, and health factors. Subsamples were visited at ages of one, three and six months and one and four years. In 2004, 2008, and 2011 when the participants had reached the mean ages of 11, 15, and 18 years, respectively, all cohort members were sought for follow-up visits. The adolescents were interviewed for behavioral and health factors and received an anthropometric evaluation at each follow-up visit. Pulmonary function tests were performed with spirometry at the 2008 and 2011 visits. All visits of the 1993 Pelotas Birth Cohort Study were approved by the Ethics Committee of the Medical School of the Federal University of Pelotas. Participants signed a written informed consent at each visit. If participants were younger than 18 years of age, a parent or guardian signed the consent form. Further details on the study design and methods have been previously described [33, 34].

### Pulmonary function

A standardized spirometry protocol conducted by a trained technician and under the supervision of an expert researcher was performed when the participants were 15 and 18 years old. The subjects were seated with their backs straight and wore a nose-clip during the tests. A battery-operated portable spirometer (Easy-One; NDD Medical Technologies, Chelmsford MA, USA and Zurich, Switzerland) was used. At least three acceptable spirometry trials were collected for each subject and the best trial was analyzed. Subjects who had undergone abdominal, eye, or thoracic surgery or had any hospital admissions in the previous three months or those with heart disease were excluded from spirometry. In addition, those who were undergoing tuberculosis treatment or who were pregnant or thought to be pregnant were also excluded. Based on these criteria, 64 and 144 adolescents were not eligible for spirometry at the 2008 and 2011 follow-up visits. In addition, 280 participants either refused spirometry testing or were lost to follow-up in 2008, while 53 participants refused spirometry testing or were lost to follow-up at the 2011 visit. The criteria of the American Thoracic Society (ATS) and European Respiratory Society (ERS) were used in order to ensure standardized high quality spirometry data [35]. Approximately 90% of the procedures for both follow-up visits reached acceptability and reproducibility standards.

Pulmonary function was assessed through FEV_1_ (forced expiratory volume in one second), FVC (forced vital capacity), and PEF (peak expiratory flow). These three pulmonary function parameters measured by spirometry at 18 years of age were the study outcomes. In the literature spirometric prediction equations have been described for different populations [36, 37]. However, these equations were not suitable for our sample. Because of this, the values of FEV_1_, FVC, and PEF were expressed as z-scores, which were generated from the standardized residuals of the study sample at 18 years of age, taking into account sex, skin color, and height at 18 years of age.

### Sedentary behavior

During the 2004, 2008, and 2011 visits, when the participants were on average 11, 15, and 18 years old, sedentary behavior was self-reported with questionnaires on the weekday frequency of television viewing, video game playing and computer use. For each screen behavior an initial question was asked regarding participation. If the answer was affirmative, a second question was asked about the time spent in each behavior on a usual weekday. For each age, sedentary behavior was calculated as the sum in hours of time spent on television viewing, video game playing and computer use. The adolescents for whom the sum was more than 12 h were excluded from the analysis in order to avoid over-reporting due to use of more than one kind of screen simultaneously. A total of 298 adolescents were excluded from the analysis based on a sum of sedentary behavior greater than 12 h in at least one follow-up visit.

To evaluate the sedentary behavior trajectory during adolescence a group-based trajectory modeling was used. This method consists of a specialized form of finite mixture modeling used to identify groups of individuals following similar progressions of a behavior or outcome over age or time [38, 39]. The three follow-up visits (at 11, 15, and 18 years of age) were used to estimate group-based trajectories. Adolescents with missing information on sedentary behavior at one or more visit were excluded. The trajectories model were estimated through Stata procedure “traj” with zero-inflated Poisson distribution [40]. The choice of the number and shape of trajectories included in the analyses was based on the best fit of the model (the maximum Bayesian information criteria, BIC, and the Akaike information criterion, AIC).

Additional analysis was performed with the cumulative sedentary behavior during adolescence. To evaluate the cumulative sedentary behavior as a outcome, a continuous variable was created through the sum of hours spent on screen-based sedentary behavior at 11, 15, and 18 years of age, and then this value was divided by three. The results of this analysis are presented in the Additional file 1.

### Statistical analysis

All statistical procedures were conducted using Stata 12.0 (StataCorp. 2011. *Stata Statistical Software: Release 12*. College Station, TX: StataCorp LP). A Chi-squared test was used to compare the sample of this study with the original birth cohort population. Crude and adjusted linear regression analyses were performed to assess the association between sedentary behavior trajectory from 11 to 18 years and pulmonary function at 18 years. Multivariate models were adjusted for skin color (self-reported) as marker of ethnicity, family income at birth, maternal schooling at birth, birth weight, smoking during pregnancy, mother’s height at birth, equivalent pulmonary function parameter at 15 years (z-score), body mass index (BMI) at 11 and 15 years, Tanner stage at 15 years [41], height at 18 years, leisure-time physical activity at 11 and 15 years, wheezing in the previous year at 18 years, and corticoid steroid use in the previous 3 months at 18 years. All analyses were stratified by sex.

## Results

The follow-up rates for the 1993 Pelotas Birth Cohort at ages 11, 15, and 18 years were 87.5%, 85.7%, and 81.3% respectively. A final sample of 3382 participants from the 1993 Pelotas Birth Cohort with complete data on sedentary behavior at ages 11, 15 and 18 years and pulmonary function at 18 years of age, were included in the current study. These individuals did not differ from the original cohort in terms of key variables (Table 1). In the multivariate analyses 2632 participants with complete information for all covariates were included. The covariates with larger number of missing information were Tanner stage at 15 years for male subjects (*n* = 195) and corticoids use in the previous 3 months at 18 years for female subjects (*n* = 114). Table 2 presents the description of sedentary behavior trajectory categories and pulmonary function parameters for the analytic sample.

**Table 1:** Characteristics of the original cohort and the sample with complete data of sedentary behavior and pulmonary function

Variable	Original Cohort - perinatal *N* (%)	Sample with exposure and outcome data *N* (%)	p*
Number of participants	5249 (100)	3382 (100)	-
Sex	5248 (100)	3382 (100)	0.291
Male	2606 (49.7)	1640 (48.5)	
Female	2642 (50.3)	1742 (51.5)	
Skin color	4323 (100)	3382 (100)	0.996
White	2769 (64.1)	2166 (64.0)	
Black	611 (14.1)	486 (14.4)	
Brown	784 (18.1)	606 (17.9)	
Yellow	76 (1.8)	61 (1.8)	
Indigenous	83 (1.9)	63 (1.9)	
Family income (quintiles)	5137 (100)	3324 (100)	0.582
1	1031 (20.1)	626 (18.9)	
2	1195 (23.2)	797 (24.0)	
3	889 (17.3)	599 (18.0)	
4	1001 (19.5)	656 (19.7)	
5	1021 (19.9)	646 (19.4)	
Maternal schooling (years of formal education)	5242 (100)	3376 (100)	0.297
0	130 (2.5)	70 (2.1)	
1–4	1338 (25.5)	847 (25.1)	
5–8	2424 (46.2)	1621 (48.0)	
≥ 9	1350 (25.8)	838 (24.8)	
Birth weight (grams)	5232 (100)	3377 (100)	0.518
< 2500	510 (9.8)	315 (9.3)	
≥ 2500	4722 (90.2)	3062 (90.7)	
Smoking during pregnancy	5249 (100)	3382 (100)	0.981
No	3497 (66.6)	2254 (66.6)	
Yes	1752 (33.4)	1128 (33.4)	

*Chi-squared test

**Table 2:** Description of sedentary behavior trajectory and pulmonary function parameters at 15 and 18 years by sex

Variable	Male	Female
Screen time trajectory from 11 to 18 years (N [%])		
Always high	690 (42.07)	631 (36.22)
Always moderate	838 (51.10)	986 (56.60)
Always low	112 (6.83)	125 (7.18)
FEV_1_ at 15 years, L (Mean [SD])	3.48 (0.66)	2.94 (0.44)
FVC at 15 years, L (Mean [SD])	4.02 (0.75)	3.30 (0.52)
PEF at 15 years, L/s (Mean [SD])	7.50 (1.43)	6.62 (1.05)
FEV_1_ at 18 years, L (Mean [SD])	4.12 (0.61)	3.04 (0.45)
FVC at 18 years, L (Mean [SD])	4.80 (0.69)	3.50 (0.51)
PEF at 18 years, L/s (Mean [SD])	8.77 (1.52)	6.48 (1.15)

FEV_1_: forced expiratory volume in 1 s; FVC: forced vital capacity; PEF: peak expiratory flow

**Fig. 1:**
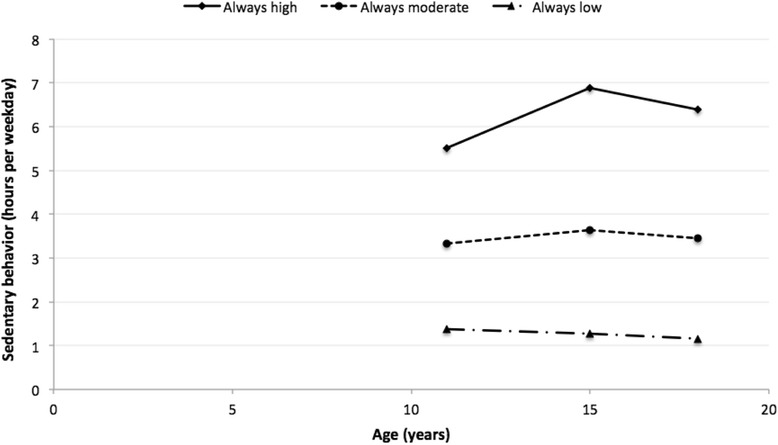
Screen-based sedentary behavior trajectories from 11 to 18 years of age

To identify trajectories for screen-based sedentary behavior, analyses were conducted specifying three-, four- and five-group models. Inspection of the adjusted model quality parameters revealed that the three-group model had the best fit. Fig. 1 shows the three-group trajectories for sedentary behavior. Group 1 (“always high”, *n* = 1379), with 38.85% of the sample, had mean hours per weekday of sedentary behavior at 11, 15 and 18 years of age of 5.51 (±0.07 SD), 6.88 (±0.07), and 6.38 (±0.07). Group 2 (“always moderate”, *n* = 1920), with 54.08% of the sample, had mean hours per weekday of sedentary behavior at 11, 15 and 18 years of 3.33 (±0.05), 3.64 (±0.05), and 3.46 (±0.05). Group 3 (“always low”, *n* = 251), with 7.07% of the sample, had mean of hours per weekday of sedentary behavior at 11, 15 and 18 years of age of 1.37 (±0.08), 1.27 (±0.07), and 1.16 (±0.07).

Table 3 shows crude and adjusted analyses for the trajectory of sedentary behavior with pulmonary function parameters. In the crude analyses, boys in the always-low group for sedentary behavior had lower FEV_1_ (β = −0.233; 95% CI:-0.457;-0.008) and PEF (β = −0.366; 95% CI:-0.588;-0.144) at 18 years compared to boys in the always-high group. Boys in the always-moderate group for sedentary behavior had lower PEF at 18 years of age (β = −0.180; 95% CI:-0.293;-0.068) compared to always-high boys in crude analyses. However, these associations did not remain significant after adjustment for potential confounders. On the other hand, boys in the always-low group for sedentary behavior had higher FVC at 18 years (β = 0.177; 95% CI:0.027;0.327) than boys in the always-high group in adjusted analysis. No significant association was found for girls in crude and adjusted analyses.

**Table 3:** Crude and adjusted analyses of sedentary behavior trajectory from 11 to 18 years of age and pulmonary function parameters at 18 years of age by sex

	Male				Female			
	Crude^a^		Adjusted^b^		Crude^a^		Adjusted^b^	
	β (95% CI)	*p*	β (95% CI)	*p*	β (95% CI)	*p*	β (95% CI)	*p*
FEV_1_ at 18 years (z-score)								
Sedentary behavior								
Always high	Ref		Ref		Ref		Ref	
Always moderate	−0.088 (−0.201;0.025)	0.126	−0.003 (−0.091;0.085)	0.953	−0.051 (−0.137;0.035)	0.245	−0.021 (−0.080;0.039)	0.494
Always low	−0.233 (−0.457;-0.008)	0.042	0.081 (−0.097;0.260)	0.373	−0.039 (−0.204;0.126)	0.645	−0.005 (−0.119;0.109)	0.928
FVC at 18 years (z-score)								
Sedentary behavior								
Always high	Ref		Ref		Ref		Ref	
Always moderate	−0.097 (−0.209;0.015)	0.088	−0.001 (−0.075;0.073)	0.980	−0.015 (−0.102;0.072)	0.742	0.012 (−0.048;0.073)	0.690
Always low	−0.159 (−0.381;0.062)	0.159	0.177 (0.027;0.327)	0.021	−0.050 (−0.217;0.117)	0.560	−0.017 (−0.132;0.099)	0.777
PEF at 18 years (z-score)								
Sedentary behavior								
Always high	Ref		Ref		Ref		Ref	
Always moderate	−0.180 (−0.293;-0.068)	0.002	−0.091 (−0.193;0.012)	0.083	−0.002 (−0.088;0.084)	0.936	0.016 (−0.060;0.092)	0.675
Always low	−0.366 (−0.588;-0.144)	0.001	−0.162 (−0.369;0.046)	0.127	0.150 (−0.016;0.316)	0.076	0.110 (−0.035;0.255)	0.137

FEV_1_: forced expiratory volume in 1 s; FVC: forced vital capacity; PEF: peak expiratory flow

^a^Crude analyses – male: *n* = 1640; female: *n* = 1742

^b^Adjusted for skin color, family income at birth, maternal schooling at birth, birth weight, smoking during pregnancy, mother’s height at birth, pulmonary function parameter at 15 years, body mass index at 11 and 15 years, Tanner stage at 15 years, leisure-time physical activity at 11 and 15 years, height at 18 years, wheezing in the previous year at 18 years, and corticoids in the previous 3 months at 18 years (male: *n* = 1243; female: *n* = 1389)

## Discussion

In our study, we evaluated the association between the trajectory of screen-based sedentary behavior during adolescence and pulmonary function at 18 years of age. This is one of the first studies on this topic and the first to evaluate the association in a young population. We did not find consistent associations between time spent on television, video games, and computers and pulmonary function at age 18 for boys and girls after adjustment for potential confounders. The only significant adjusted association was found between sedentary behavior and FVC in boys; those boys with lower sedentary behavior showed higher values of FVC at 18 years.

Studies evaluating the relationship between screen-based sedentary behavior and pulmonary function are scarce in the literature. The only study to our knowledge that evaluated the association between sedentary behavior and pulmonary function was carried out in individuals from 45 to 74 years of age [42]. The authors of this study did not find association between television viewing and change in FEV_1_. However, comparing to our results is difficult since we evaluated sedentary behavior trajectory during adolescence rather than adulthood. To date there have been no other longitudinal studies evaluating this relationship in young populations. We found that boys with lower sedentary behavior during adolescence had higher FVC at 18 years compared to boys with higher sedentary behavior. Since this was the only significant association found after adjustment for potential confounders, we cannot refuse the possibility of residual confounding.

The study of the consequences of sedentary behavior on health is a relatively new paradigm in the physical activity field [43]. There is no consensus yet in the literature regarding the association between physical activity and pulmonary function. Some longitudinal studies have shown a positive association in children and adolescents [44, 45], and in adults [46]. One of these studies was carried out with the same population as in the current study [45]. Although sedentary behavior may be related to physical activity, the association between them in young individuals is weak, suggesting that these behaviors do not necessarily displace one another [47]. Sedentary behavior is not the opposite of physical activity; they are different behaviors with independent determinants [48]. Despite of no consistent association was found between more time spent in sedentary behavior and reduced pulmonary function, spending excessive time in sedentary behavior may have negative consequences on health outcomes. A meta-analysis showed that for children and adolescents there is strong evidence of association between sedentary behavior and obesity and moderate evidence for the association between sedentary behavior and blood pressure, total cholesterol, physical fitness, self-esteem, and social behavior problems [9].

The mechanisms through which physical activity and sedentary behavior might impact pulmonary function remain unclear. A possible explanation may be that low physical activity levels and excessive sedentary behavior are associated with lower level of physical fitness or increased body size [19, 49], resulting in reduced pulmonary function. A recent study showed that spending more time in sedentary behavior per day was associated with lower cardiorespiratory fitness, independent from higher intensity physical activity [49]. Cross-sectional studies have found a positive association between physical fitness and higher values of pulmonary function [50, 51]. Bae et al. [51] found a significant positive correlation between muscle strength and power with FEV_1_ and FVC values in students aged 6–17 years. Also, Lazarus et al. [50] found that percentage body fat was negatively associated with FVC in adults, while fat-free mass and muscle strength showed a positive association. Moreover, sedentary activity, such as television viewing, has been associated with consumption of high-density foods [52, 53] and obesity [9, 13]. Excess fat mass may impair ventilatory mechanics because of the stiffness of the thoracic cage due to fat accumulation around the ribs, abdomen and diaphragm [54] or even due to systemic inflammation that may cause airway inflammation and a consequent alteration in pulmonary function [55]. Thus, the possible association between sedentary behavior and pulmonary function might be explained by this mediating effect of obesity and fat distribution [42]. Another possible explanation of the association between screen-based sedentary behavior and pulmonary function may be the different respiratory patterns during sedentary activities. There is some evidence that prolonged periods of watching videotapes is associated with a decreased frequency of spontaneous sighs, a physiologic phenomenon that helps regulate airway tone [56]. More research on this is needed to better understand these relationships in a longitudinal way.

Our study has several strengths. It was carried out in a large population-based sample with high rates of retention and follow-up, minimizing the likelihood of selection bias. The longitudinal design allows the assessment of temporality between sedentary behavior and later adolescent pulmonary function. In addition, the sedentary behavior trajectory was evaluated through group-based trajectory modeling, a robust method used to identify groups of individuals with similar developmental trajectories [38, 39]. Approximately 90% of the spirometric test data in the study reached international quality criteria [35]. As noted earlier, our study is the first to evaluate the association between screen-based sedentary behavior and pulmonary function in adolescents.

The major limitation of our study is the self-reported screen-based sedentary behavior data. Self-reported data may lead to misclassification or bias, and might reduce the chance of identifying associations. However, objective measures have limitations as well, and questionnaires are widely used to measure sedentary behavior in population-based studies [57]. Another limitation for measuring and analyzing sedentary behavior is the lack of consensus in the literature regarding cut-point for classifying an individual as sedentary [58]. The trajectory groups used in our analyses might not adequately represent categories with differential risk. However, we also analyzed the outcome as a continuous variable and the results were similar (Additional file 1: Table S1). The possibility of over-reporting the time spent on screen activities due to the use of more than one kind of screen at the same time may also be a limitation. In order to minimize this problem, we excluded from the analysis those adolescents who reported extremely high numbers of hours of sedentary behavior.

## Conclusion

In conclusion, this study did not find consistent association between screen-based sedentary behavior trajectory during adolescence and pulmonary function at age 18 among participants from a birth cohort study in Brazil. These findings show that spending excessive time in sitting activities such as television viewing, video game playing or computer use may not impair pulmonary function in youth. Since to the best of our knowledge this is the first study to examine the association between sedentary behavior and pulmonary function in adolescents, further investigation on this subject is needed. Studies evaluating sedentary behavior by objective measures and investigating the possible mediators, as well as the possible influence of physical activity and obesity in the relationship between sedentary behavior and pulmonary function are important to provide more information on this issue.

## Additional file

Additional file 1: Table S1. Crude and adjusted analyses of average cumulative sedentary behavior from 11 to 18 years of age and pulmonary function parameters at 18 years of age by sex. (DOCX 64 kb)

## Abbreviations

FEV_1_: Forced expiratory volume in 1 s
FVC: Forced vital capacity
PEF: Peak expiratory flow

## References

[1] Pate RR, O'Neill JR, Lobelo F (2008). The evolving definition of "sedentary". Exerc Sport Sci Rev.

[2] Owen N, Healy GN, Matthews CE, Dunstan DW (2010). Too much sitting: the population health science of sedentary behavior. Exerc Sport Sci Rev.

[3] Sisson SB, Church TS, Martin CK, Tudor-Locke C, Smith SR, Bouchard C (2009). Profiles of sedentary behavior in children and adolescents: the US National Health and nutrition examination survey, 2001-2006. Int J Pediatr Obes.

[4] Hallal PC, Andersen LB, Bull FC, Guthold R, Haskell W, Ekelund U (2012). Global physical activity levels: surveillance progress, pitfalls, and prospects. Lancet.

[5] Oliveira JS, Barufaldi LA, Abreu Gde A, Leal VS, Brunken GS, Vasconcelos SM (2016). ERICA: use of screens and consumption of meals and snacks by Brazilian adolescents. Rev Saude Publica.

[6] Pate RR, Mitchell JA, Byun W, Dowda M (2011). Sedentary behaviour youth. Br J Sports Med.

[7] Mielke GI, da Silva IC, Owen N, Hallal PC (2014). Brazilian adults' sedentary behaviors by life domain: population-based study. PLoS One.

[8] WHO (2009). Global health risks: mortality and burden of disease attributable to selected major risks.

[9] de Rezende LF, Rodrigues Lopes M, Rey-Lopez JP, Matsudo VK, Luiz OC (2014). Sedentary behavior and health outcomes: an overview of systematic reviews. PLoS One.

[10] Dunstan DW, Barr EL, Healy GN, Salmon J, Shaw JE, Balkau B (2010). Television viewing time and mortality: the Australian diabetes, obesity and lifestyle study (AusDiab). Circulation.

[11] Grontved A, Hu FB (2011). Television viewing and risk of type 2 diabetes, cardiovascular disease, and all-cause mortality: a meta-analysis. JAMA.

[12] Chau JY, Grunseit AC, Chey T, Stamatakis E, Brown WJ, Matthews CE (2013). Daily sitting time and all-cause mortality: a meta-analysis. PLoS One.

[13] Tremblay MS, LeBlanc AG, Kho ME, Saunders TJ, Larouche R, Colley RC (2011). Systematic review of sedentary behaviour and health indicators in school-aged children and youth. Int J Behav Nutr Phys Act.

[14] Wilmot EG, Edwardson CL, Achana FA, Davies MJ, Gorely T, Gray LJ (2012). Sedentary time in adults and the association with diabetes, cardiovascular disease and death: systematic review and meta-analysis. Diabetologia.

[15] Ekelund U, Steene-Johannessen J, Brown WJ, Fagerland MW, Owen N, Powell KE (2016). Does physical activity attenuate, or even eliminate, the detrimental association of sitting time with mortality? A harmonised meta-analysis of data from more than 1 million men and women. Lancet.

[16] Tsai HJ, Tsai AC, Nriagu J, Ghosh D, Gong M, Sandretto A (2007). Associations of BMI, TV-watching time, and physical activity on respiratory symptoms and asthma in 5th grade schoolchildren in Taipei, Taiwan. J Asthma.

[17] Vogelberg C, Hirsch T, Radon K, Dressel H, Windstetter D, Weinmayr G (2007). Leisure time activity and new onset of wheezing during adolescence. Eur Respir J.

[18] Corbo GM, Forastiere F, De Sario M, Brunetti L, Bonci E, Bugiani M (2008). Wheeze and asthma in children: associations with body mass index, sports, television viewing, and diet. Epidemiology.

[19] Sherriff A, Maitra A, Ness AR, Mattocks C, Riddoch C, Reilly JJ (2009). Association of duration of television viewing in early childhood with the subsequent development of asthma. Thorax.

[20] Groth SW, Rhee H, Kitzman H (2016). Relationships among obesity, physical activity and sedentary behavior in young adolescents with and without lifetime asthma. J Asthma.

[21] Vlaski E, Stavric K, Seckova L, Kimovska M, Isjanovska R (2008). Influence of physical activity and television-watching time on asthma and allergic rhinitis among young adolescents: preventive or aggravating?. Allergol Immunopathol (Madr).

[22] Arvaniti F, Priftis KN, Papadimitriou A, Yiallouros P, Kapsokefalou M, Anthracopoulos MB (2011). Salty-snack eating, television or video-game viewing, and asthma symptoms among 10- to 12-year-old children: the PANACEA study. J Am Diet Assoc.

[23] Mitchell EA, Beasley R, Bjorksten B, Crane J, Garcia-Marcos L, Keil U (2013). The association between BMI, vigorous physical activity and television viewing and the risk of symptoms of asthma, rhinoconjunctivitis and eczema in children and adolescents: ISAAC phase three. Clin Exp Allergy.

[24] Jones SE, Merkle SL, Fulton JE, Wheeler LS, Mannino DM (2006). Relationship between asthma, overweight, and physical activity among U.S. high school students. J Community Health.

[25] Priftis KN, Panagiotakos DB, Anthracopoulos MB, Papadimitriou A, Nicolaidou P (2007). Aims, methods and preliminary findings of the physical activity, nutrition and allergies in children examined in Athens (PANACEA) epidemiological study. BMC Public Health.

[26] Kim JW, So WY, Kim YS (2012). Association between asthma and physical activity in Korean adolescents: the 3rd Korea youth risk behavior web-based survey (KYRBWS-III). Eur J Pub Health.

[27] Vaccaro JA, Niego J, Huffman FG (2016). Dietary factors, body weight, and screen time in U.S. children with and without asthma. Children's Health Care.

[28] Menezes AM, Wehrmeister FC, Muniz LC, Perez-Padilla R, Noal RB, Silva MC (2012). Physical activity and lung function in adolescents: the 1993 Pelotas (Brazil) birth cohort study. J Adolesc Health.

[29] Twisk JW, Staal BJ, Brinkman MN, Kemper HC, van Mechelen W (1998). Tracking of lung function parameters and the longitudinal relationship with lifestyle. Eur Respir J.

[30] Knuiman MW, James AL, Divitini ML, Ryan G, Bartholomew HC, Musk AW (1999). Lung function, respiratory symptoms, and mortality: results from the Busselton health study. Ann Epidemiol.

[31] Menezes AM, Perez-Padilla R, Wehrmeister FC, Lopez-Varela MV, Muino A, Valdivia G (2014). FEV1 is a better predictor of mortality than FVC: the PLATINO cohort study. PLoS One.

[32] Meszaros D, Dharmage SC, Matheson MC, Venn A, Wharton CL, Johns DP (2010). Poor lung function and tonsillectomy in childhood are associated with mortality from age 18 to 44. Respir Med.

[33] Victora CG, Araujo CL, Menezes AM, Hallal PC, Vieira Mde F, Neutzling MB (2006). Methodological aspects of the 1993 Pelotas (Brazil) birth cohort study. Rev Saude Publica.

[34] Goncalves H, Assuncao MC, Wehrmeister FC, Oliveira IO, Barros FC, Victora CG (2014). Cohort profile update: the 1993 Pelotas (Brazil) birth cohort follow-up visits in adolescence. Int J Epidemiol.

[35] Miller MR, Hankinson J, Brusasco V, Burgos F, Casaburi R, Coates A (2005). Standardisation of spirometry. Eur Respir J.

[36] Pereira CAC (2002). Espirometria. J Pneumol.

[37] Quanjer PH, Stanojevic S, Cole TJ, Baur X, Hall GL, Culver BH (2012). Multi-ethnic reference values for spirometry for the 3-95-yr age range: the global lung function 2012 equations. Eur Respir J.

[38] Nagin DS (2005). Group-based modeling of development.

[39] Nagin DS, Odgers CL (2010). Group-based trajectory modeling in clinical research. Annu Rev Clin Psychol.

[40] Jones BL, Nagin DS (2012). A Stata plugin for estimating group-based trajectory models.

[41] Tanner JM (1962). Growth at adolescence.

[42] Jakes RW, Day NE, Patel B, Khaw KT, Oakes S, Luben R (2002). Physical inactivity is associated with lower forced expiratory volume in 1 second : European prospective investigation into cancer-Norfolk prospective population study. Am J Epidemiol.

[43] Katzmarzyk PT (2010). Physical activity, sedentary behavior, and health: paradigm paralysis or paradigm shift?. Diabetes.

[44] Ji J, Wang SQ, Liu YJ, He QQ (2013). Physical activity and lung function growth in a cohort of Chinese school children: a prospective study. PLoS One.

[45] da Silva BGC, Wehrmeister FC, Quanjer PH, Perez-Padilla R, Goncalves H, Horta BL (2016). Physical activity in early adolescence and pulmonary function gain from 15 to 18 years of age in a birth cohort in Brazil. J Phys Act Health.

[46] Pelkonen M, Notkola IL, Lakka T, Tukiainen HO, Kivinen P, Nissinen A (2003). Delaying decline in pulmonary function with physical activity: a 25-year follow-up. Am J Respir Crit Care Med.

[47] Pearson N, Braithwaite RE, Biddle SJ, van Sluijs EM, Atkin AJ (2014). Associations between sedentary behaviour and physical activity in children and adolescents: a meta-analysis. Obes Rev.

[48] Van Der Horst K, Paw MJ, Twisk JW, Van Mechelen W (2007). A brief review on correlates of physical activity and sedentariness in youth. Med Sci Sports Exerc.

[49] van der Velde JH, Koster A, van der Berg JD, Sep SJ, van der Kallen CJ, Dagnelie PC, et al. Sedentary behavior, physical activity, and fitness-the Maastricht study. Med Sci Sports Exerc. 2017; [Epub ahead of print] 10.1249/MSS.0000000000001262 28319587

[50] Lazarus R, Gore CJ, Booth M, Owen N (1998). Effects of body composition and fat distribution on ventilatory function in adults. Am J Clin Nutr.

[51] Bae JY, Jang KS, Kang S, Han DH, Yang W, Shin KO (2015). Correlation between basic physical fitness and pulmonary function in Korean children and adolescents: a cross-sectional survey. J Phys Ther Sci.

[52] Blass EM, Anderson DR, Kirkorian HL, Pempek TA, Price I, Koleini MF (2006). On the road to obesity: television viewing increases intake of high-density foods. Physiol Behav.

[53] Wiecha JL, Peterson KE, Ludwig DS, Kim J, Sobol A, Gortmaker SL (2006). When children eat what they watch: impact of television viewing on dietary intake in youth. Arch Pediatr Adolesc Med.

[54] Naimark A, Cherniack RM (1960). Compliance of the respiratory system and its components in health and obesity. J Appl Physiol.

[55] Scott HA, Gibson PG, Garg ML, Pretto JJ, Morgan PJ, Callister R (2012). Relationship between body composition, inflammation and lung function in overweight and obese asthma. Respir Res.

[56] Hark WT, Thompson WM, McLaughlin TE, Wheatley LM, Platts-Mills TA (2005). Spontaneous sigh rates during sedentary activity: watching television vs reading. Ann Allergy Asthma Immunol.

[57] Atkin AJ, Gorely T, Clemes SA, Yates T, Edwardson C, Brage S (2012). Methods of measurement in epidemiology: sedentary behaviour. Int J Epidemiol.

[58] Guerra PH, Farias Junior JC, Florindo AA (2016). Sedentary behavior in Brazilian children and adolescents: a systematic review. Rev Saude Publica.

