# Supplementary material for: Screen-based sedentary behavior during adolescence and pulmonary function in a birth cohort
Source: Int J Behav Nutr Phys Act. 2017 Jun 23;14:82. doi: 10.1186/s12966-017-0536-5 (PMC5481971; doi:10.1186/s12966-017-0536-5)
Supplement: Additional file 1: Table S1. — Crude and adjusted analyses of average cumulative sedentary behavior from 11 to 18 years of age and pulmonary function parameters at 18 years of age by sex. (DOCX 64 kb) [file 12966_2017_536_MOESM1_ESM.docx]

| **Supplementary Table 1.** Crude and adjusted analyses of average cumulative sedentary behavior from 11 to 18 years of age and pulmonary function parameters at 18 years of age by sex. | | | | | | | | |
| --- | --- | --- | --- | --- | --- | --- | --- | --- |
|  | **Male** | | | | **Female** | | | |
|  | **Crude*** | | **Adjusted**** | | **Crude*** | | **Adjusted**** | |
|  | **β (95%CI)** | **p** | **β (95%CI)** | **p** | **β (95%CI)** | **p** | **β (95%CI)** | **p** |
| **FEV_1_ at 18 years (z-score)** | 0.037 (0.010;0.067) | 0.008 | 0.002 (-0.021;0.025) | 0.861 | 0.016 (-0.007;0.039) | 0.167 | 0.003 (-0.013;0.019) | 0.730 |
| **FVC at 18 years (z-score)** | 0.033 (0.005;0.061) | 0.023 | -0.001 (-0.021;0.018) | 0.888 | 0.011 (-0.012;0.035) | 0.346 | 0.001(-0.016;0.017) | 0.993 |
| **PEF at 18 years (z-score)** | 0.062 (0.022;0.090) | <0.001 | 0.033 (0.006;0.059) | 0.015 | -0.009 (-0.033;0.014) | 0.431 | -0.007 (-0.028;0.014) | 0.501 |
| FEV_1_: forced expiratory volume in 1 second; FVC: forced vital capacity; PEF: peak expiratory flow.  *Crude analyses – male: n=1640; female: n=1742  **Adjusted for skin color, family income at birth, maternal schooling at birth, birth weight, smoking during pregnancy, mother’s height at birth, pulmonary function parameter at 15 years, body mass index at 11 and 15 years, Tanner stage at 15 years, leisure-time physical activity at 11 and 15 years, height at 18 years, wheezing in the previous year at 18 years, and corticoids in the previous 3 months at 18 years (male: n=1243; female: n=1389). | | | | | | | | |
